# BIO-LGCA: A cellular automaton modelling class for analysing collective cell migration

**DOI:** 10.1371/journal.pcbi.1009066

**Published:** 2021-06-15

**Authors:** Andreas Deutsch, Josué Manik Nava-Sedeño, Simon Syga, Haralampos Hatzikirou

**Affiliations:** 1 Centre for Information Services and High Performance Computing, Technische Universität Dresden, Dresden, Germany; 2 Department of Mathematics, Universidad Nacional Autónoma de México, Mexico City, Mexico; 3 Department of Mathematics, Khalifa University, Abu Dhabi, United Arab Emirates; Inria, FRANCE

## Abstract

Collective dynamics in multicellular systems such as biological organs and tissues plays a key role in biological development, regeneration, and pathological conditions. Collective tissue dynamics—understood as population behaviour arising from the interplay of the constituting discrete cells—can be studied with on- and off-lattice agent-based models. However, classical on-lattice agent-based models, also known as cellular automata, fail to replicate key aspects of collective migration, which is a central instance of collective behaviour in multicellular systems. To overcome drawbacks of classical on-lattice models, we introduce an on-lattice, agent-based modelling class for collective cell migration, which we call biological lattice-gas cellular automaton (BIO-LGCA). The BIO-LGCA is characterised by synchronous time updates, and the explicit consideration of individual cell velocities. While rules in classical cellular automata are typically chosen ad hoc, rules for cell-cell and cell-environment interactions in the BIO-LGCA can also be derived from experimental cell migration data or biophysical laws for individual cell migration. We introduce elementary BIO-LGCA models of fundamental cell interactions, which may be combined in a modular fashion to model complex multicellular phenomena. We exemplify the mathematical mean-field analysis of specific BIO-LGCA models, which allows to explain collective behaviour. The first example predicts the formation of clusters in adhesively interacting cells. The second example is based on a novel BIO-LGCA combining adhesive interactions and alignment. For this model, our analysis clarifies the nature of the recently discovered invasion plasticity of breast cancer cells in heterogeneous environments.

## Author summary

Pattern formation during embryonic development and pathological tissue dynamics, such as cancer invasion, emerge from individual intercellular interactions. In order to study the impact of single cell dynamics and cell-cell interactions on tissue behaviour, one needs to develop space-time-dependent on- or off-lattice agent-based models (ABMs), which consider the behaviour of individual cells. However, classical on-lattice agent-based models also known as cellular automata fail to replicate key aspects of collective migration, which is a central instance of collective behaviour in multicellular systems. Here, we present the rule- and lattice-based BIO-LGCA modelling class which allows for (i) rigorous derivation of rules from biophysical laws and/or experimental data, (ii) mathematical analysis of collective migration, and (iii) computationally efficient simulations.

## Introduction

Systems biology and mathematical modelling is rapidly expanding its scope from the study of single cells to the analysis of collective behaviour in multicellular tissue- and organ-scale systems. In such systems, individual cells may interact with their environment (hapto- and chemotaxis, contact guidance, etc.) or with other cells (cell-cell adhesion, contact inhibiton of locomotion, etc.) and produce collective patterns exceeding the cells’ interaction range. To study collective behaviour in such systems theoretically and/or computationally, a mathematical model must be decided upon as a first step. State-continuous models describe the dynamics of cell densities. Their lack of resolution at the individual scale makes them inappropriate to investigate the role of individuals in collective behaviour. Agent-based models, on the other hand, are particularly suited to the study of collective behaviour in multicellular systems, as they resolve individual cell dynamics, and thus allow for the analysis of large-scale tissue effects of individual cell behaviour.

Various agent-based models have been developed to analyse tissue dynamics as a collective phenomenon emerging from the interplay of individual biological cells. In these models, cells are regarded as separate, individual units, contrary to continuum models, which neglect the discrete individual cell nature, and where tissue dynamics is derived from conservation and constitutive laws, drawing parallels to physical systems. Since agent-based models represent individual biological cells, distinct cell phenotypes can be taken into account, which may be fundamental for analysing the organisation at the tissue level. For example, it has been shown that cell-to-cell variability plays a key role in tumour progression and resistance to treatment [1]. Moreover, with the advance of high performance computing, agent-based models can be used to analyse *in vitro* systems at a 1:1 basis even for large cell population sizes.

Agent-based models can be classified into on-lattice and off-lattice models depending on whether or not cell movement is restricted to an underlying lattice (see [2] for references). Various off-lattice models exist to study different types of single and collective cell migration [3–8].

In lattice models, either (i) a lattice site may be occupied by many biological cells (e.g., [9]), (ii) a site may be occupied by at most one single biological cell, or (iii) several neighbouring lattice sites may represent a single biological cell (e.g., [10]). Model types (i) and (ii) can mimic volume exclusion effects, (iii) can qualitatively capture cell deformation and compression, while each of the three approaches can describe the effects of mechanical forces of one cell on its neighbour, or on a group of neighbouring cells to some extent.

Lattice models are equivalent to cellular automata (CA), which were introduced by J. v. Neumann and S. Ulam in the 1950s as models for individual self-reproduction [11]. A cellular automaton consists of a regular spatial lattice in which each lattice node can assume a discrete, typically finite number of states. The next state of a node solely depends on the states in neighbouring sites and a deterministic or stochastic transition function. One distinguishes CA with synchronous and asynchronous update. Cellular automata provide simple models of self-organising systems in which collective behaviour emerges from an ensemble of interacting “simple” components—being it molecules, cells or organisms [12–14]. The interacting particle system (IPS) is an example of probabilistic CA with asynchronous update. Here, proliferation, death, and migration of biological cells are modeled as stochastic processes.

However, when modelling collective migration phenomena, classical CA and, particularly, IPS models have major drawbacks which are due to the strict volume exclusion and asynchronous update. Most importantly, these models fail to reproduce collective movement at unit density, since volume exclusion at high densities results in a “jammed state”. However, a fluidised state at unit density is an important case of collective migration especially in epithelial tissues. Furthermore, the asynchronous update in IPS models may lead to oscillating density spikes. For example, in an IPS model for persistent motion in a crowded environment, cells at the invasion front detach and leave gaps behind that are subsequently filled by following cells [15]. This is an artefact of the asynchronous update since invasion typically happens while cells stay connected. Moreover, classic CA models consider only cell position and not explicitly cell momentum, complicating the modelling of collective cell migration mediated primarily through changes in momentum, rather than density [16].

The lattice-gas cellular automaton (BIO-LGCA) introduced here is a cellular automaton in which lattice sites are updated synchronously, and which explicitly considers individual cell velocities. These features make the BIO-LGCA appropriate for modelling collective migration phenomena where cell interactions result in directional changes of velocity, and where high cell densities do not hamper movement.

**Fig 1:**
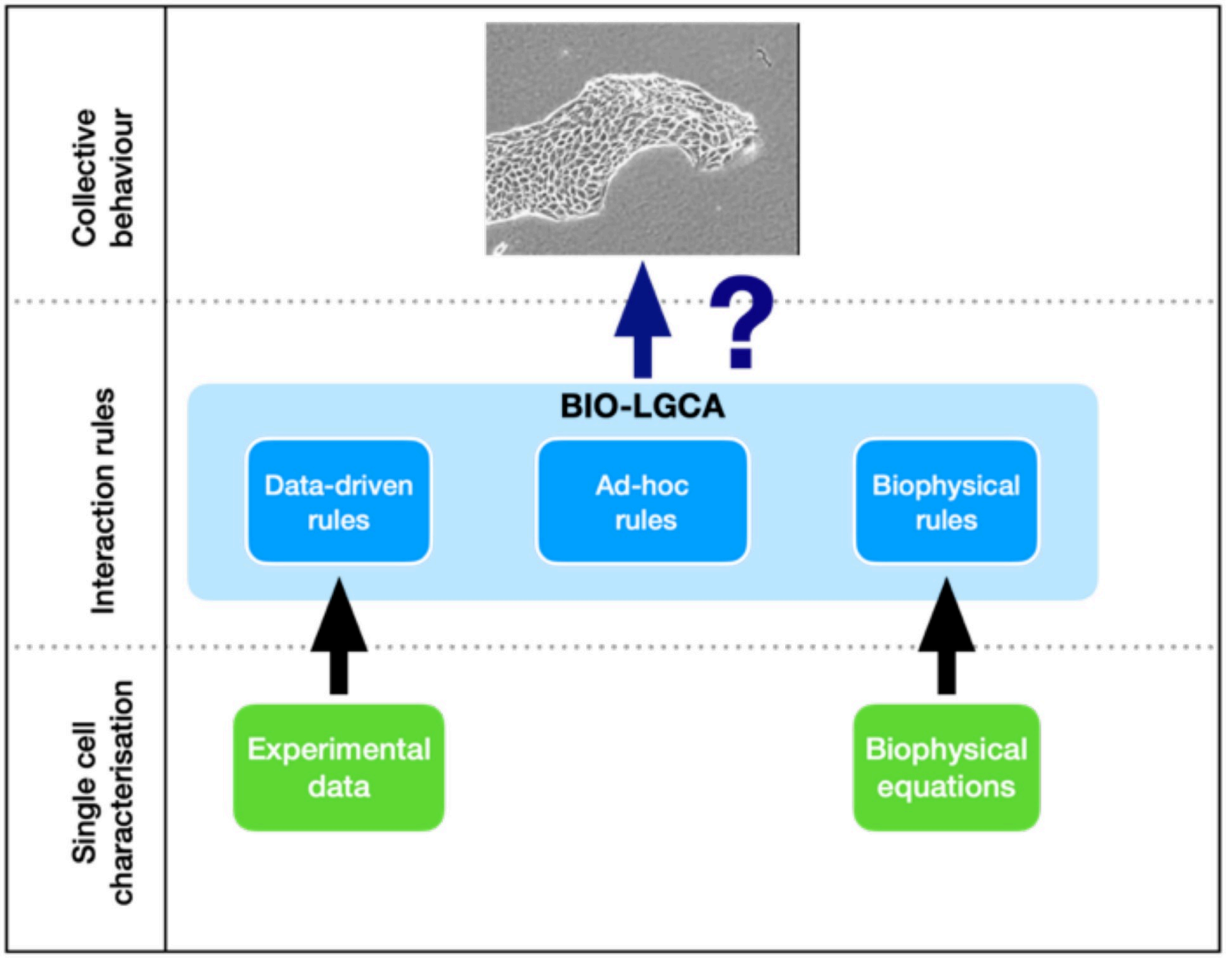
BIO-LGCA modelling. The key question is to identify the interaction rules underlying a particular collective phenomenon in a population of cells. BIO-LGCA interaction rules can be chosen ad hoc, extracted from experimental single cell migration data, or derived from biophysical equations for single cell migration.

The structure of the paper is as follows: we first formally define the BIO-LGCA model class. Then, we construct *biophysical BIO-LGCA rules* from microscopic Langevin models for selected cases of single and collective cell migration. Subsequently, we demonstrate how to generate *data-driven BIO-LGCA rules* from experimental single cell migration data (Fig 1). Furthermore, we show that, in specific cases, the biophysical and the data-driven approaches converge to the same functional form. For this case, we introduce several biologically relevant model examples. Finally, we present two examples of mean-field analysis. The first example allows to predict the formation of cluster patterns in simulations of a BIO-LGCA model with adhesive cell-cell interaction. The second example analyses a novel BIO-LGCA combining adhesive interactions and alignment to explain the recently discovered invasion plasticity of breast cancer cells in heterogeneous environments. We end with a critical discussion of the BIO-LGCA modelling framework.

## Materials and methods

A BIO-LGCA is defined by a discrete spatial lattice L, a discrete state space E, a neighbourhood N and local rule-based dynamics.

### Lattice

**Fig 2:**
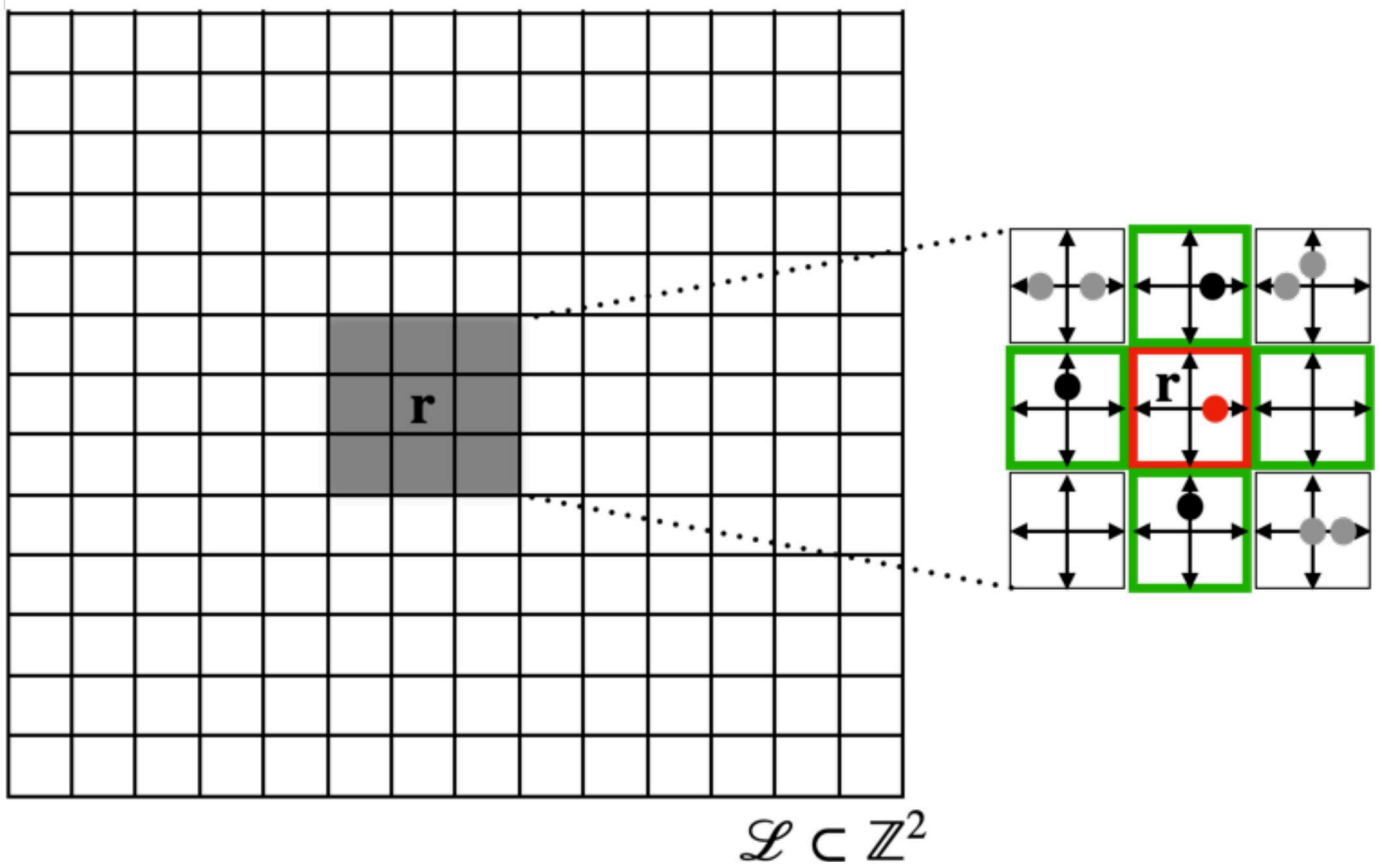
Lattice and neighbourhood in the BIO-LGCA. Example of square lattice (left). The node state is represented by the occupation of velocity channels (right); in the example, there are four velocity channels **c**_1_, **c**_2_, **c**_3_, **c**_4_, corresponding to the lattice directions, and one “rest channel” **c**_5_. Filled dots denote the presence of a cell in the respective velocity channel; right: von Neumann neighbourhood (green) of the red node. Black dots represent interaction partners of the red dot. Gray dots lie outside of the interaction neighbourhood and do not interact with the red dot.

The regular lattice $L\subset R^{d}$ consists of nodes r∈L. Every node has *b* nearest-neighbours, where *b* depends on the lattice geometry. Each lattice node r∈L is connected to its nearest neighbours by unit vectors **c**_*i*_, *i* = 1, …, *b*, called velocity channels. In addition, a variable number $a\in N_{0}$ of rest channels (zero-velocity channels) $c_{j}=0,\;b<j\leq a+b,$ is allowed (Fig 2). The parameter *K* = *a* + *b* defines the maximum *node capacity*.

### Neighbourhood

The set N, the neighbourhood template, defines the nodes which determine the dynamics of the node 0∈L. Throughout this work, the neighbourhood will be assumed to be a von Neumann neighbourhood (Fig 2), defined as $N^{b}≔N^{b}\left(0\right)=\left\{c_{1},c_{2},\ldots ,c_{b}\right\}$, but other neighbourhood choices are possible. In general, $N\left(r\right)≔N^{b}\left(r\right)=N^{b}+r=\left\{r+r^{{}^{\prime }},r^{{}^{\prime }}\in N^{b}\right\}$, specifies the set of lattice nodes which determine the dynamics of the state at node r∈L.

### State space

The state space in LGCA is defined through the occupation numbers *s*_*j*_ ∈ {0, 1}, *j* = 1, …, *K*. These occupation numbers represent the presence (*s*_*j*_ = 1) or absence (*s*_*j*_ = 0) of a cell in the channel **c**_*j*_ within some node. Then, the configuration of a node is given by the state vector

$$\begin{array}{c} s=(s_{1},\ldots ,s_{K})\in E={\left\{0,1\right\}}^{K}. \end{array}$$

This reflects an *exclusion principle* which allows not more than one cell at the same node within the same channel simultaneously. As a consequence, each node r∈L can host up to *K* cells, which are distributed in different channels.

It is possible to consider more than one cell phenotype in the BIO-LGCA model. In this case each phenotype is indexed by σ∈Σ⊂N. Then, the configuration vector is given by

$$\begin{array}{c} s={\left(s_{\sigma }\right)}_{\sigma \in \Sigma }\in E={\left\{0,1\right\}}^{|\Sigma |K}, \end{array}$$

where |⋅| denotes the cardinality of a set. Each node will be able to support up to |Σ|*K* cells.

Two useful quantities for a configuration **s** at a given node are the *total number of cells at the node* *n*(**s**) and the *momentum/node flux* **J**(**s**), defined as

$$\begin{array}{c} n\left(s\right)≔\sum_{\sigma \in \Sigma }\;n_{\sigma }\left(s\right)=\sum_{\sigma \in \Sigma }\sum_{j=1}^{K}\;s_{\sigma ,j\;}\;\text{and}\;J\left(s\right)≔\sum_{\sigma \in \Sigma }\sum_{j=1}^{K}\;s_{\sigma ,j\;}c_{j}, \end{array}$$

where *n*_*σ*_(**s**) is the *σ* number of cell phenotypes.

### Dynamics

In general, in cellular automata a new lattice configuration is created according to a local rule that determines the new state of each node in terms of the current states of the node and the nodes in its neighbourhood. In order to determine a new lattice configuration, the local rule is applied independently and simultaneously at every node **r** of the lattice. Mathematically, in probabilistic cellular automata, the local rule can be interpreted as a transition probability *P*(**s** → **s**′) to replace a current configuration **s** by a new node configuration **s**′.

**Fig 3:**
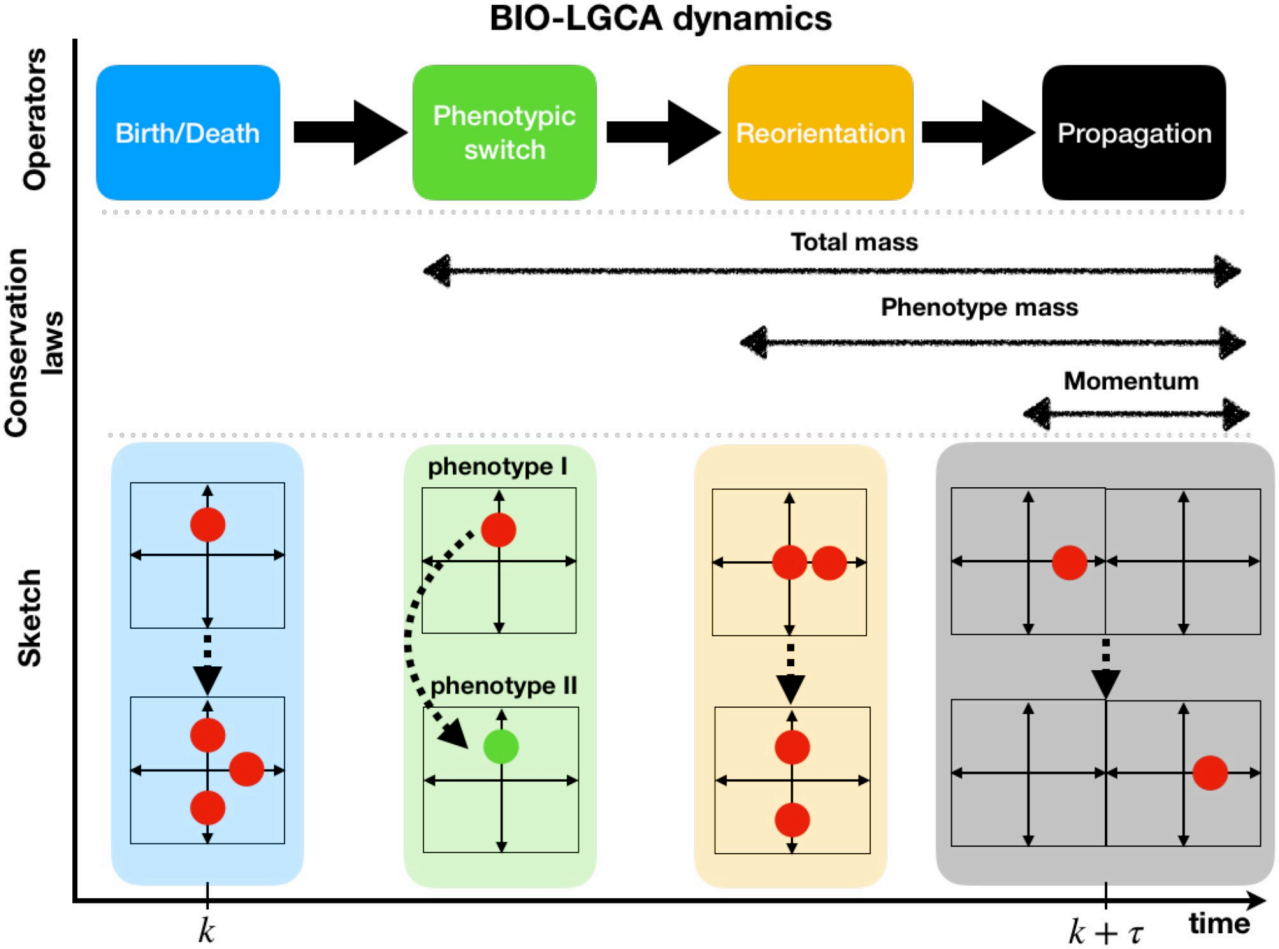
Operator-based dynamics of the BIO-LGCA. Propagation P, reorientation O, phenotypic switch S, and birth/death operators R (top); conservation laws maintained by the different operators (middle); sketches of the operator dynamics (bottom), see text for explanations.

In a BIO-LGCA, local rules are composed of a combination of operators for stochastic reorientation (O), phenotypic switching (S), and stochastic cell birth and death (R), as well as a deterministic propagation operator (P) (see Fig 3). The propagation and reorientation operators together define cell movement, while phenotypic switching allows cells to stochastically and reversibly transition between phenotypes. In a BIO-LGCA, the stochastic operators are applied sequentially to every node, such that the transition probability can be expressed as

$$\begin{array}{c} P\left(s\to s^{\prime }\right)=P_{S}P_{R}P_{O}, \end{array}$$

where $P_{i},i\in \left\{S,R,O\right\}$ are the transition probabilities of the corresponding operator. In this way, a post-interaction node configuration **s**′ is defined as the resulting node configuration after subsequent application of the stochastic operators, i.e. $s^{\prime }=s^{S\circ R\circ O}$. Subsequently, the deterministic propagation operator P is applied: cells occupying velocity channels at the node, i.e. moving cells, are translocated to neighbouring nodes in the direction of their respective velocity channels. The time step increases once the propagator operator has been applied. Accordingly, the dynamics of the BIO-LGCA can be summarised by the stochastic microdynamical equation

$$\begin{array}{c} s_{j}(r+c_{j},k+1)=s_{j}^{\prime }(r,k). \end{array}$$ (1)

## Results

### BIO-LGCA rule derivation

**Fig 4:**
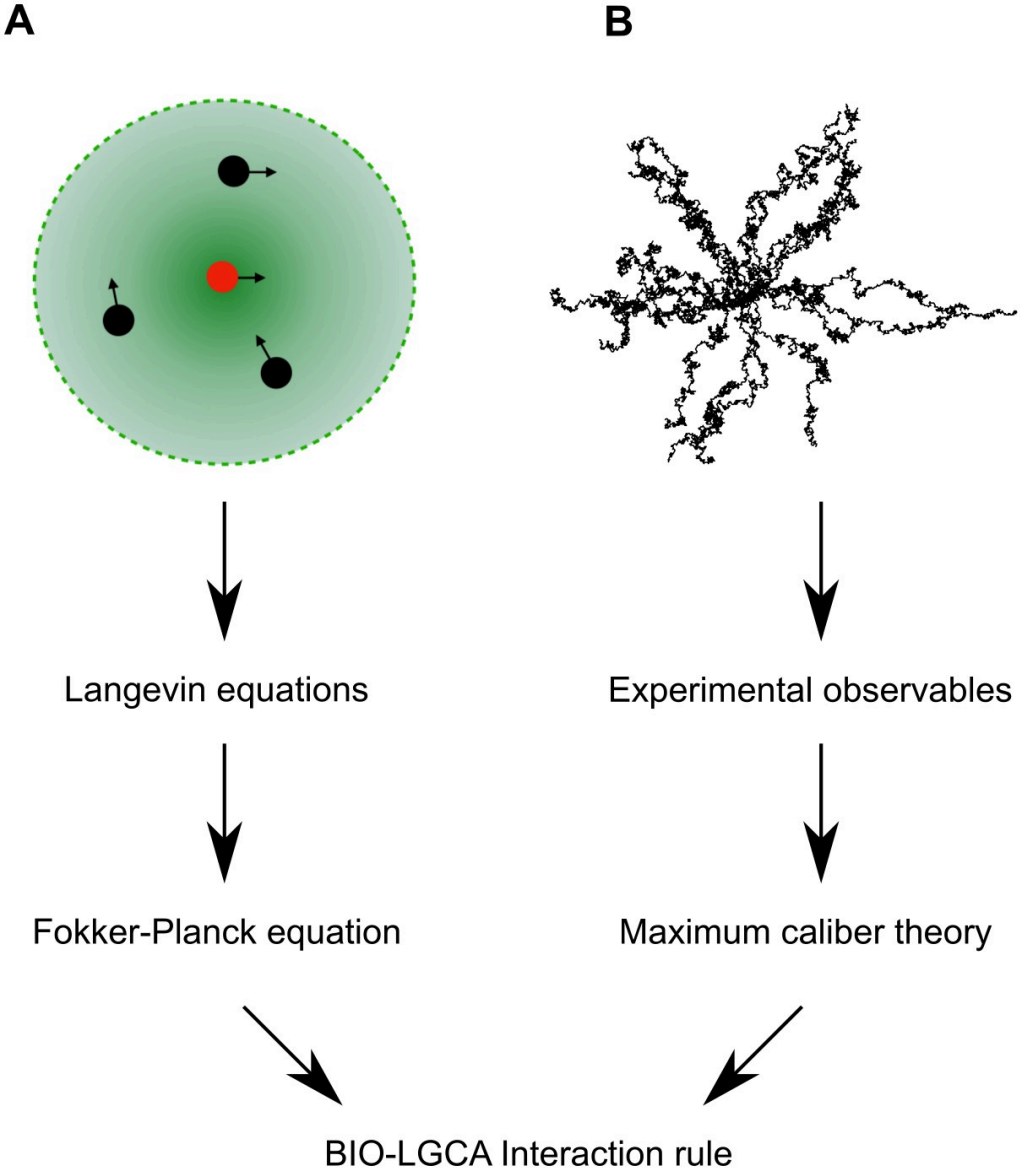
Rule generation in BIO-LGCA models. A: Starting from the Langevin equations of a self-propelled particle model, the interaction rule can be obtained from the steady-state distribution of the associated Fokker-Planck equation. B: Alternatively, experimental observables can be used as input for the maximum caliber theory to derive the probabilities of cell tracks and the corresponding interaction rule.

In classical cellular automata, transition probabilities are typically chosen *ad hoc*. Here, we show that BIO-LGCA rules can also be derived from biophysical equations of motion and from experimental data. In the following, we disregard birth/death processes and phenotypic transitions. Accordingly, the corresponding BIO-LGCA model is specified exclusively by its reorientation probability (cp. subsection “Dynamics” and Fig 3). We first present a method to derive the BIO-LGCA reorientation probability from a Langevin model for cell migration [17]. A similar procedure has also been used to derive rules for CA models describing molecular movement in crowded environments [18]. Secondly, we sketch a method to obtain the reorientation probability from experimental observations [19]. In certain cases, independent of the applied rule derivation method, the functional form of the transition probability will be the same (Fig 4).

#### Reorientation dynamics derived from biophysical equations of motion

It has been shown that various types of cell migration can be described by self-propelled particle models (SPPs). These off-lattice models are defined by a set of stochastic differential equations governing the motion of discrete cells in an overdamped situation, e.g in a highly viscous medium. The stochastic differential equations encoding individual cell motion (as introduced in [20]) are Langevin equations, where a stochastic variable *θ*_*m*_ describes the orientation of the *m*-th cell in the system which moves with a constant speed $v_{0}\in R^{+}$ and orientation *θ*_*m*_(*t*) ∈ [0, 2*π*) varying according to a given interaction potential and influenced by noise. The Langevin equations of motion read [21]

$$\begin{array}{cc} {\dot{x}}_{m} & =v_{0}v\left(\theta_{m}\right), \end{array}$$ (2a)

$$\begin{array}{cc} {\dot{\theta }}_{m} & =-\gamma \frac{\partial U(\{x_{k}\},\{\theta_{k}\})}{\partial \theta_{m}}+\xi_{m}\left(t\right), \end{array}$$ (2b)

where $x_{m}\in R^{d}$ is the cell’s spatial position, $v\left(\theta_{m}\right)\in R^{d}$ is a unit vector pointing in the direction of the cell’s displacement, $\gamma \in R^{+}$ is a relaxation constant, and *ξ*_*m*_(*t*) is a white noise term with zero mean and correlation 〈*ξ*_*m*_(*t*_1_) *ξ*_*n*_(*t*_2_)〉 = 2*D*_*θ*_ *δ*(*t*_2_ − *t*_1_)*δ*_*m*,*n*_. The heart of the model is the potential $U(\{x_{k}\},\{\theta_{k}\}):R^{Nd}\times {\left[0,2\pi \right)}^{N}\mapsto R$, where *N* is the number of cells within the *m*−th cell’s neighbourhood of interaction, and {**x**_*k*_} and {*θ*_*k*_} are the sets of all neighbouring cells’ positions and orientations, respectively. This potential encodes the biophysical mechanisms that dictate the cell’s reorientation. Here, we assume that the reorientation potential only depends on the orientations of neighbouring cells, though a dependence on cell positions is also possible [22].

The probability density function of the stochastic variable *θ*_*m*_ governed by the Langevin equations is given by the Fokker-Planck equation

$$\begin{array}{c} \frac{\partial P(\theta_{m},t)}{\partial t}=\gamma \frac{\partial }{\partial \theta_{m}}(\frac{\partial U}{\partial \theta_{m}}P(\theta_{m},t))+D_{\theta }\frac{\partial^{2}P(\theta_{m},t)}{\partial \theta_{m}^{2}}. \end{array}$$ (3)

If we assume fast relaxation times for the solution of the Fokker-Planck equation, then one can choose the stationary solution as the probability of cell *m* to have an orientation *θ*_*m*_, i.e.

$$\begin{array}{c} P\left(\theta_{m}\right)=C_{0}\;\text{exp}\left(-\beta U\left(\theta_{m}\right)\right), \end{array}$$ (4)

where *β* = *γ*/*D*_*θ*_ and *C*_0_ is an integration constant. Applying a discretisation of the particle orientations and under the assumption that the relaxation time of the Fokker-Planck solution is smaller than the BIO-LGCA time step and that the channel occupations in the LGCA model are independent one can derive the reorientation probability

$$\begin{array}{c} \begin{array}{cc} P(s\to s^{O})= & \frac{\delta (n(s),n(s^{O}))}{Z}\prod_{j=1}^{b}{\left\{C_{0}\;\text{exp}[-\beta U(c_{j})]\right\}}^{s_{j}^{O}}\\ & =\frac{\delta (n(s),n(s^{O}))}{\tilde{Z}}\text{exp}\;\{-\beta \sum_{j=1}^{b}[U\left(c_{j}\right)s_{j}^{O}]\}, \end{array} \end{array}$$ (5)

where $Z=\sum_{s^{O}}\prod_{j=1}^{b}P{\left(c_{j}\right)}^{s_{j}^{O}}$ and $\tilde{Z}$ is a normalisation constant in which the integration constant *C*_0_ has been absorbed [17].

#### Collective migration

SPP models for collective cell migration often use interaction potentials of the following form

$$\begin{array}{c} U(\{x_{k}\},\{\theta_{k}\})=-C(\{x_{k}\},\{\theta_{k}\})\;\text{cos}\;[\theta_{m}-\varphi (\{x_{k}\},\{\theta_{k}\})], \end{array}$$ (6)

where amplitude *C* (interaction strength) and shift *φ* (optimal orientation) may depend on the positions and/or orientations of all cells within the neighbourhood of interaction (including the central cell). Using trigonometric identities, the reorientation potential can then be rewritten as

$$\begin{array}{c} U(\{x_{k}\},\{\theta_{k}\})=-v(\theta_{m})\cdot G(\{x_{k}\},\{\theta_{k}\}), \end{array}$$ (7)

where **G**({**x**_*k*_}, {*θ*_*k*_}) is called the *local director field*, whose norm and argument are, respectively, ‖**G**({**x**_*k*_}, {*θ*_*k*_})‖ = *C*({**x**_*k*_}, {*θ*_*k*_}) and arg [**G**({**x**_*k*_}, {*θ*_*k*_})] = *φ*({**x**_*k*_}, {*θ*_*k*_}). Substituting Eq 7 in Eq 5, and using the linearity of the internal product, the transition probability of the reorientation operator is

$$\begin{array}{c} P(s\to s^{O})=\frac{\delta (n(s),n(s^{O}))}{Z}\text{exp}\;(\beta J(s)\cdot G(s_{N})), \end{array}$$ (8)

where $G(s_{N})$ is the local director field of the neighbourhood configuration, and **J**(**s**) is the node flux, as described previously.

More generally, whenever the reorientation potential can be expressed as

$$\begin{array}{c} U(\{x_{k}\},\{\theta_{k}\})=-C(\{x_{k}\},\{\theta_{k}\})\;{\text{cos}}^{n}\;[\theta_{m}-\varphi (\{x_{k}\},\{\theta_{k}\})], \end{array}$$ (9)

with n∈N, the argument of the exponential in the transition probability can be expressed as an internal product of two vectors. In the specific case of *n* = 2, using trigonometric functions, one arrives at the transition probability

$$\begin{array}{c} P(s\to s^{O})=\frac{\delta (n(s),n(s^{O}))}{Z}\text{exp}\;(\beta N(s)\cdot G(s_{N})), \end{array}$$ (10)

where **N**(**s**) is the *local nematic alignment vector*, which is defined as

$$\begin{array}{c} N(s)≔\frac{1}{2}\sum_{p=1}^{b}c_{[2(p-1)\text{mod}b]+1}s_{p}. \end{array}$$

Thus, the reorientation probabilities have the same general form whenever the interaction potential is conservative (i.e. time-independent) and consists of a pairwise comparison between the angles and/or positions of neighbouring cells.

#### Data-driven rules

Besides from Langevin equations defining SPP models, it is also possible to derive BIO-LGCA reorientation probabilities from experimental data (Fig 4). For this, we assume that certain observables, e.g. an autocorrelation function, have been obtained from primary migration data. Then, we can use the maximum caliber (or maximum path entropy) formalism [23] to obtain the most unbiased probability distribution of paths that reproduces the experimental observables. This translates into maximising the following functional

$$\begin{array}{c} \tilde{C}[P_{\Gamma }]=-\sum_{\Gamma }P_{\Gamma }\;\text{ln}\;P_{\Gamma }+\sum_{j=1}^{k}\beta \left(j\right)[\sum_{\Gamma }P_{\Gamma }{\tilde{U}}_{j}({\left\{s(r)\right\}}_{r\in L})-E_{j}]+\lambda (\sum_{\Gamma }P_{\Gamma }-1), \end{array}$$

where *P*_Γ_ is the probability of a cell to follow a certain spatial trajectory Γ, *β*(*j*) and λ are Lagrange multipliers, ${\tilde{U}}_{j}({\left\{s(r)\right\}}_{r\in L})$ is the value of the optimised observable at time step *j* depending on the configuration of the lattice ${\left\{s(r)\right\}}_{r\in L}$, and *E*_*j*_ is the value of the experimental observable at time step *j*. The first term of the functional is the entropy, which we want to maximise. The second term restricts the resulting probabilities to match the experimental observation. Since we assume the experimental observable, *E*_*j*_ to be a time-dependent function, a Lagrange multiplier *β*(*j*) is needed for every time step *j*. The last term guarantees the normalisation of probabilities, which requires an additional Lagrange multiplier, λ.

The optimisation of this functional yields an optimal value for the path probabilities $P_{\Gamma }=\frac{1}{Z}\text{exp}[\sum_{j=1}^{k}\beta \left(j\right){\tilde{U}}_{j}({\left\{s(r)\right\}}_{r\in L})]$, where the value of *β*(*j*) is such that $E_{k}=\sum_{\Gamma }P_{\Gamma }{\tilde{U}}_{j}({\left\{s(r)\right\}}_{r\in L})$.

If the process is Markovian, then one may decompose the path probability into individual channel occupation probabilities for each time step *k*, as

$$\begin{array}{c} P_{i,k}=\frac{1}{Z}\text{exp}\;[\beta \left(k\right){\tilde{U}}_{k}(s_{i}|s_{N})], \end{array}$$ (11)

where *Z* is a normalisation constant and the optimised observable is dependent on the occupancy of the *i*-th channel of the node and conditioned to a certain configuration of its interaction neighbourhood. For example, if the observation is the autocorrelation function *g*(*t*) = 〈**v**_0_⋅**v**_*t*_〉, where **v**_*t*_ denotes the normalised velocity of a cell at time *t*, determined from experimental data, then the corresponding channel occupation probabilities are found to be

$$\begin{array}{c} P_{i,k}=\frac{1}{z}\text{exp}\;[dg\left(k\right)(c_{i_{0}}\cdot c_{i})], \end{array}$$ (12)

where *z* is the normalisation constant for the transition probability, *d* is the dimension of space, and $c_{i_{0}}$ is the initial orientation of the cell.

If we assume independence among cells within the same node, we arrive at a reorientation probability of the form

$$\begin{array}{c} P(s\to s^{O})=\frac{\delta (n(s),n(s^{O}))}{Z}\text{exp}\;[\sum_{i=1}^{b}\beta \left(k\right){\tilde{U}}_{k}(s_{i}|s_{N})]. \end{array}$$ (13)

Note that, if both the experimental observation and optimised observable are time independent, then the Lagrange multiplier *β*(*k*) = *β* is also time independent, and the transition probabilities are given by

$$\begin{array}{c} P(s\to s^{O})=\frac{\delta (n(s),n(s^{O}))}{Z}\text{exp}\;[\sum_{i=1}^{b}\beta \tilde{U}(s_{i}|s_{N})]. \end{array}$$ (14)

Furthermore, if $\tilde{U}(s_{i}|s_{N})=s_{i}c_{i}\cdot G(s_{N})$, then Eq 14 reduces to Eq 8.

In conclusion, we can construct rules of the BIO-LGCA directly from experimental observables and the structure of these rules is the same as in our ad-hoc and SPP model-derived rules.

### BIO-LGCA rules for single and collective cell migration

Here, we present key examples of transition probabilities corresponding to reorientation operators, which model important elementary single-cell and collective behaviours. Note that several of these examples’ transition probabilities have the general form of Eq 8.

**Random walk**. Random walks are performed by cells such as bacteria and amoebae in the absence of any environmental cues. Random walk of cells can be modeled by a reorientation operator with the following transition probabilities:

$$\begin{array}{c} P(s\to s^{O})=\frac{1}{Z(s)}\cdot \delta (n\left(s\right),n\left(s^{O}\right)). \end{array}$$ (15)

This rule conserves mass, i.e. cell number.

**Chemotaxis**. Chemotaxis describes the dependence of individual cell movement on a chemical signal gradient field. Accordingly, spatio-temporal pattern formation at the level of cells and chemical signals can be observed. Chemotactic patterns result from the coupling of different spatio-temporal scales at the cell and the molecular level, respectively.

To mimic a chemotactic response to the local signal concentration, we define the signal gradient field

$$\begin{array}{c} G_{\text{sig}}\left(s_{N}\right)≔\sum_{p=1}^{b}c_{p}\;c_{sig}^{p},\;s_{N}=(\left(s^{1},c_{sig}^{1}\right),\ldots ,\left(s^{b},c_{sig}^{b}\right))\in {\bar{E}}^{N}, \end{array}$$ (16)

where ${\bar{E}}^{N}=E\times R_{0}^{+}$. Chemotaxis can be modeled through a reorientation operator with transition probabilities given by

$$\begin{array}{c} P(s\to s^{O}|s_{N};\beta )=\frac{1}{Z(s_{N},\beta )}\;\text{exp}\;(\beta \;G_{\text{sig}}\left(s_{N}\right)\cdot J\left(s^{O}\right))\delta_{n\left(s\right),n\left(s^{O}\right)}. \end{array}$$ (17)

where *β* is the *chemotactic sensitivity* of the cells.

With large probability, cells will move in the direction of the external chemical gradient **G**_sig_.

**Haptotaxis**. We consider cell migration in a static environment that conveys directional information expressed by a vector field

$$\begin{array}{c} E:L\to R^{2}. \end{array}$$

A biologically relevant example is haptotactic cell motion of cells responding to fixed local concentration differences of adhesion molecules along the extracellular matrix (ECM). In this example, the local spatial concentration differences of integrin ligands in the ECM constitute a gradient field that creates a “drift” **E** [24].

The transition probabilities associated to the reorientation operator, given a vector $E\in R^{2}$, is given by

$$\begin{array}{c} P(s\to s^{O}|E)=\frac{1}{Z(n(s),E,\beta )}\text{exp}\;(\beta E\cdot J(s^{O}))\cdot \delta_{n\left(s\right),n\left(s^{O}\right)}, \end{array}$$

where $E\in R^{2}$.

In this case, cells preferably move in the direction of the external gradient **E**.

**Contact guidance**. We now focus on cell migration in environments that convey orientational, rather than directional, guidance. Examples of such motion are provided by neutrophil or leukocyte movement through the pores of the ECM, the motion of cells along fibrillar tissues, or the motion of glioma cells along fiber track structures. Such an environment can be represented by a second rank tensor field that encodes the spatial anisotropy along the tissue. In each point, the corresponding tensor informs the cells about the local orientation and strength of the anisotropy and induces a principal (local) axis of movement. Thus, the environment can again be represented by a vector field

$$\begin{array}{c} E:L\to R^{2}. \end{array}$$

Contact guidance can be modeled through a reorientation operator with transition probabilities defined as

$$\begin{array}{c} P(s\to s^{O}|E)=\frac{1}{Z(n(s),E,\beta )}\text{exp}\;(\beta |E\cdot J(s^{O})|)\cdot \delta_{n\left(s\right),n\left(s^{O}\right)}. \end{array}$$

#### Collective cell migration

**Fig 5:**
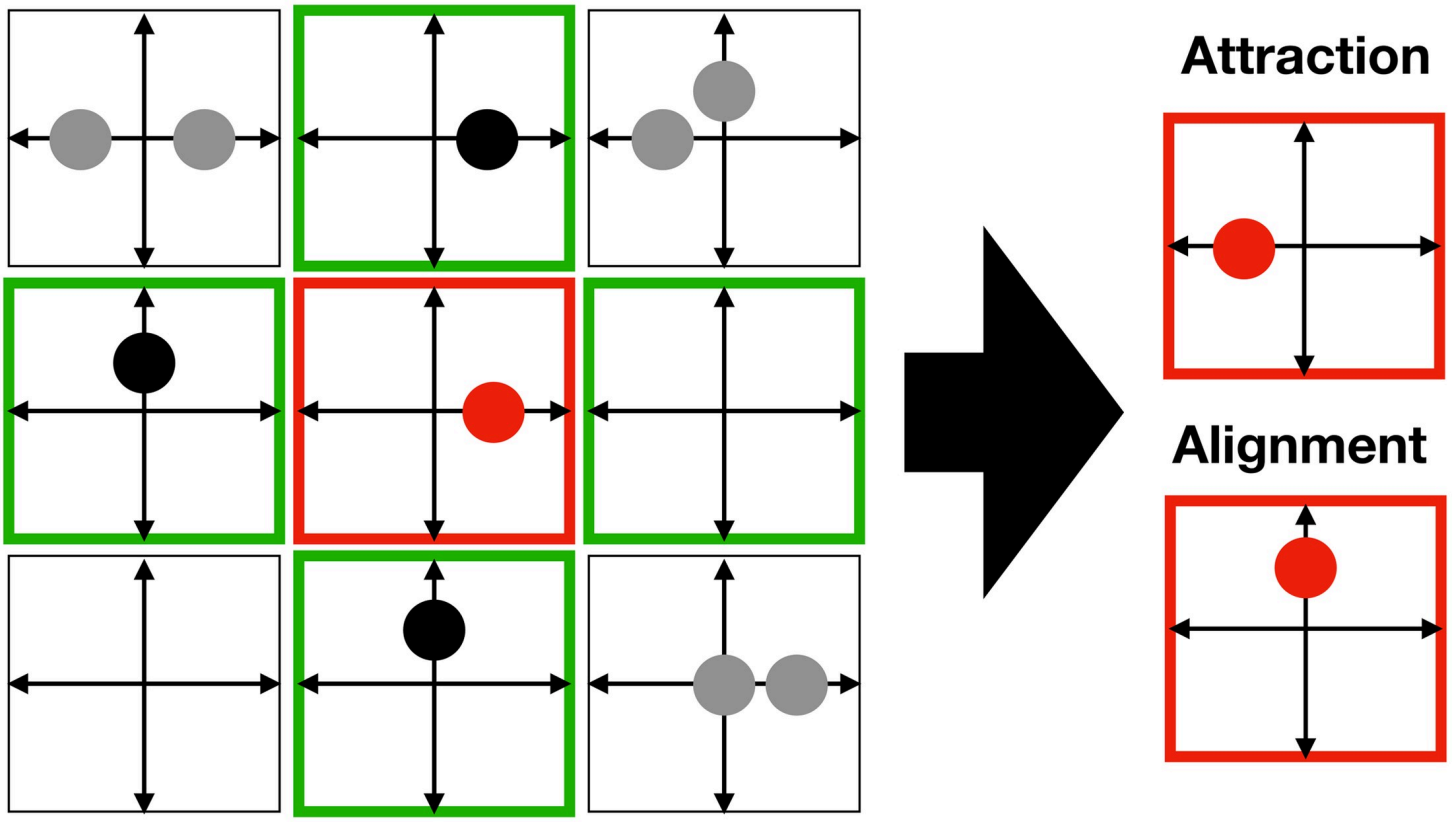
Basic interactions with neighbourhood impact. Node configuration (red) before and after application of stochastic interaction rule: cell-cell attraction (top), polar cell alignment (bottom). Gray dots represent cells outside the interaction neighbourhood.

Several kinds of organisms, as well as biological cells, e.g. fibroblasts, can align their velocities globally through local interactions. Here, we introduce a reorientation operator where the local director field is a function of the states of several channels and nodes, reflecting the influence of neighbouring cells during collective cell migration (Fig 5).

$$\begin{array}{c} P(s,\to s^{O}|s_{N})=\frac{1}{Z(s_{N})}\text{exp}\;(\beta \;D\left(s_{N}\right)\cdot J\left(s^{O}\right))\delta (n\left(s\right),n\left(s^{O}\right)), \end{array}$$ (18)

where $D\left(s_{N}\right)=\sum_{p=1}^{b}J\left(s^{p}\right)$ is the *local cell momentum*. This particular reorientation probability triggers polar cell alignment [25].

#### Attractive interaction

Biological cells can interact via cell-cell adhesion, through cadherin interaction along filopodia, for example. Agent attraction/adhesion can be modeled with a reorientation operator with the following probability distribution.

$$\begin{array}{c} P(s,\to s^{O}|s_{N},\beta )=\frac{1}{Z(s_{N})}\text{exp}\;(\beta \;G\left(s_{N}\right)\cdot J\left(s^{O}\right))\delta (n\left(s\right),n\left(s^{O}\right)), \end{array}$$ (19)

where $G\left(s_{N}\right)=\sum_{p=1}^{K}n\left(s^{p}\right)c_{p}$ is the *density gradient field*.

This reorientation probability favors cell agglomeration (Fig 5). A similar rule has been introduced in [26].

### Mean-field analysis of a BIO-LGCA model with attractive interaction

We here demonstrate the mean-field analysis of the BIO-LGCA model for attractive interaction (cp. Eq (19)). This analysis allows to predict collective behaviour in the form of cell aggregation. In particular, we calculate the critical sensitivity *β*_*c*_, such that aggregation occurs for *β* > *β*_*c*_, while a homogeneous initial condition is stable for *β* < *β*_*c*_. Under “mean-field” we understand that we neglect correlations between the occupation numbers of different channels and that we approximate the mean value of any function *f* of a random variable *X* by the function evaluated at the mean value of the random variable, i.e. 〈*f*(*X*)〉 ≈ *f*(〈*X*〉). As we are interested in the onset of aggregation from a homogeneous initial state with low density $\bar{\rho }≔\frac{1}{\left|L\right|}\sum_{r\in L}n\left(r\right)\ll 1$ and weak interaction *β* ≪ 1 we can linearise the transition probabilities. We further assume that there is at most one cell at each node and therefore only consider single cell transitions (*n* = 1). For the partition function *Z* we then obtain

$$\begin{array}{c} Z=\sum_{i=1}^{K}\text{exp}\;\beta G\left(s_{N}\right)\cdot c_{i}\approx \sum_{i=1}^{K}1+\beta G\left(s_{N}\right)\cdot c_{i}=K, \end{array}$$ (20)

due to the lattice symmetry. For the single cell transition probability we obtain

$$\begin{array}{c} P\left(s_{i}^{O}=1|s_{N}\right)\approx \frac{1+\beta G\left(s_{N}\right)\cdot c_{i}}{K}. \end{array}$$ (21)

Since the transition probability only depends on the number of cells at the neighbouring nodes, but not on their distribution on the channels, we analyse the mean local density

$$\begin{array}{c} \rho \left(r,k\right)≔\langle n(r,k)\rangle =\langle \sum_{i=1}^{K}s_{i}\left(r,k\right)\rangle . \end{array}$$ (22)

According to the propagation rule, the cell number *n*(*r*, *k* + 1) is given by

$$\begin{array}{c} n\left(r,k+1\right)=\sum_{i=1}^{K}s_{i}^{O}\left(r-c_{i},k\right). \end{array}$$ (23)

We calculate the expected value under the mean-field assumption in terms of numbers of cells *n*(**r**) at node r∈L, and the number of cells $n_{N\left(r\right)}$ in the neighbourhood of r∈L as

$$\begin{array}{c} \rho \left(r,k+1\right)=\sum_{i=1}^{K}\sum_{n_{N}}P(s_{i}^{O}=1|n_{N(r-c_{i})})P(n_{N(r-c_{i})}). \end{array}$$ (24)

As in the low-density regime P(n(r)>1)≈0∀r∈L, we can use the single-cell transition probability Eq (21) and the factorising probability distribution under our mean-field assumption to obtain

$$\begin{array}{cc} \rho (r,k+1) & =\sum_{i=1}^{K}\sum_{n_{N}}\frac{1+\beta c_{i}\cdot \sum_{j=1}^{K}c_{j}n(r-c_{i}+c_{j},k)}{K}P(n_{N(r-c_{i})})\\ & \approx \frac{1}{K}\sum_{i=1}^{K}[1+\beta c_{i}\cdot \sum_{j=1}^{b}c_{j}\rho (r-c_{i}+c_{j},k)]P(n(r-c_{i})=1)\\ & \approx \frac{1}{K}\sum_{i=1}^{K}[1+\beta c_{i}\cdot \sum_{j=1}^{b}c_{j}\rho (r-c_{i}+c_{j},k)]\rho (r-c_{i},k). \end{array}$$ (25)

To proceed, we assume a one-dimensional lattice, where *b* = 2, *c*_1,2_ = ±1 with *a* rest channels to obtain the finite-difference equation (FDE)

$$\begin{array}{c} \rho \left(r,k+1\right)=\frac{a}{K}\rho \left(r,k\right)+\frac{1}{K}[\rho (r-1,k)+\rho (r+1,k)]+\\ \frac{\beta }{K}\{\rho \left(r-1,k\right)[\rho (r,k)-\rho (r-2,k)]+\rho \left(r+1,k\right)[\rho (r,k)-\rho (r+2,k)]\}. \end{array}$$ (26)

This FDE can be analysed by means of linear stability analysis. To do this, we first rewrite Eq (26) in terms of the density difference Δ*ρ*(*r*, *k*) ≔ *ρ*(*r*, *k* + 1) − *ρ*(*r*, *k*), which we linearise around the steady state for a small perturbation of the form $\rho \left(r,k\right)=\bar{\rho }+\delta \rho \left(r,k\right)$

$$\begin{array}{cc} \Delta \rho (r,k) & \approx {\Delta \rho (r,k)|}_{\bar{\rho }}+\sum_{r^{\prime }}{\frac{\partial \Delta \rho (r,k)}{\partial \rho (r^{\prime },k)}|}_{\bar{\rho }}\delta \rho \left(r^{\prime },k\right) \end{array}$$ (27)

$$\begin{array}{c} =K^{-1}\{\delta \rho \left(r,k\right)[-2+2\beta \bar{\rho }]\\ +[\delta \rho (r+1,k)+\delta \rho (r-1,k)]\\ -\left[\delta \rho \left(r+2,k\right)+\delta \rho \left(r-2,k\right)\right]\beta \bar{\rho }\}. \end{array}$$ (28)

We now apply the discrete Fourier transform

$$\begin{array}{c} \delta \tilde{\rho }\left(q,k\right)=\sum_{r=0}^{L-1}\delta \rho \left(r,k\right)\;\text{exp}\frac{-2\pi iqr}{L}, \end{array}$$ (29)

and obtain the mode-dependent FDE

$$\begin{array}{cc} \Delta \tilde{\rho }\left(q,k+1\right) & =\frac{2\delta \tilde{\rho }\left(q,k\right)}{K}\{\beta \bar{\rho }-1+\text{cos}\frac{2\pi q}{L}-\beta \bar{\rho }\;\text{cos}\frac{4\pi q}{L}\} \end{array}$$ (30)

$$\begin{array}{cc} & =\frac{2\delta \tilde{\rho }\left(q,k\right)}{K}\{2\beta \bar{\rho }-1+\text{cos}\frac{2\pi q}{L}-2\beta \bar{\rho }\;{\text{cos}}^{2}\frac{2\pi q}{L}\}, \end{array}$$ (31)

using 2 cos *x* = *e*^*ix*^ + *e*^−*ix*^ and cos 2*x* = 2 cos ^2^ *x* − 1. Note that the system becomes unstable when the r.h.s. of the equation is larger than 0, meaning the perturbation grows, while it is stable with a decreasing perturbation if it is smaller than 0. To find the dominant Fourier mode *q* that maximises the r.h.s. we assume an infinite lattice *L* → ∞ such that we can define the quasi-continuous wave number $\kappa ≔\frac{2\pi q}{L}$ and use the derivative with respect to *κ* to calculate the maxima of the bracket on the r.h.s. of Eq (31),

$$\begin{array}{cc} -\text{sin}\;\kappa +4\beta \bar{\rho }\;\text{cos}\;\kappa \;\text{sin}\;\kappa & =0, \end{array}$$ (32)

$$\begin{array}{cc} -1+4\beta \bar{\rho }\;\text{cos}\;\kappa & =0, \end{array}$$ (33)

$$\begin{array}{cc} \text{cos}\;\kappa & =\frac{1}{4\beta \bar{\rho }}. \end{array}$$ (34)

**Fig 6:**
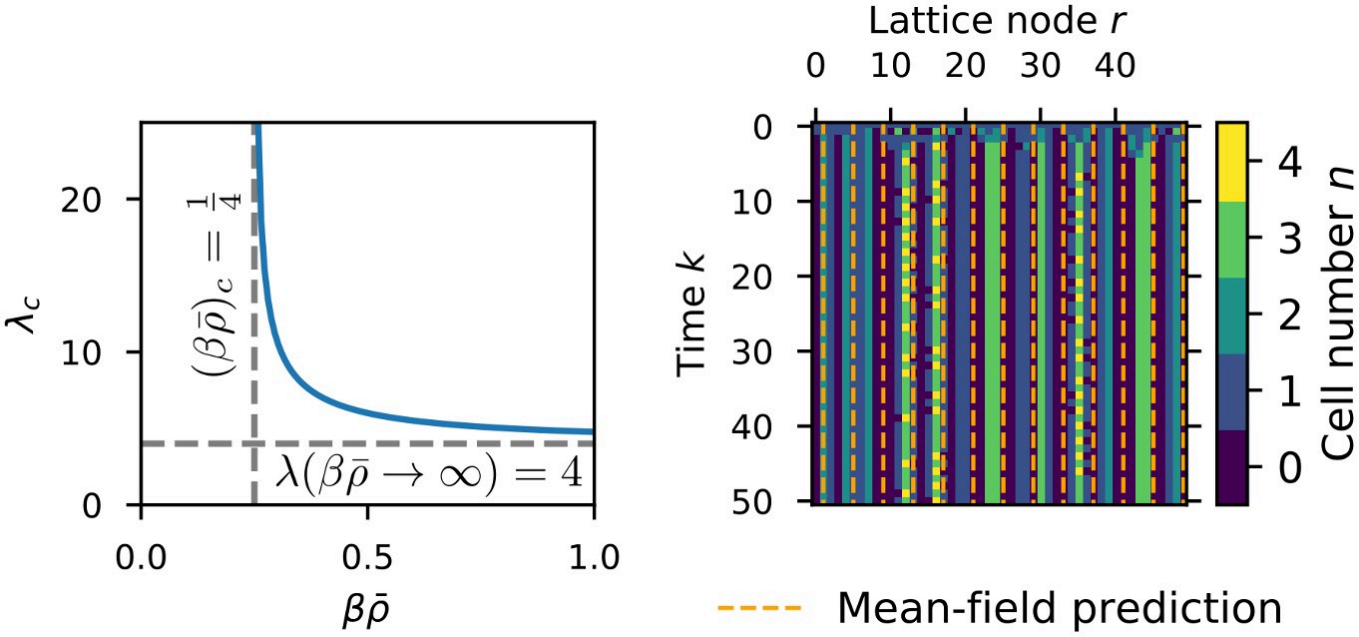
Pattern formation in the LGCA aggregation model. Left: critical wave length obtained from the mean-field analysis. The critical wave length diverges for $\left(\beta \bar{\rho }\right)\to {\left(\beta \bar{\rho }\right)}_{c}=1/4$, and ${\text{lim}}_{{\left(\beta \bar{\rho }\right)}_{c}\to \infty }\lambda_{c}=4$. Right: emergence of a periodic pattern from a homogeneous initial state. The horizontal distance of the orange dashed lines is equal to the critical wave length predicted by mean-field analysis. Parameters: $\beta =100,\bar{\rho }=1,a=2$.

Note that we divided by sin *κ* here, neglecting the trivial solutions *κ* = 0, *π*. Clearly the solution $\text{cos}\;\kappa_{c}=\frac{1}{4\beta \bar{\rho }}$ is only valid for $\beta \bar{\rho }\geq \frac{1}{4}$ and it is the dominant wave number in this case. This in turn allows us to define the critical parameter combination ${\left(\beta \bar{\rho }\right)}_{c}≔\frac{1}{4}$. We can also calculate the dominant wave length in dependence of $\beta \bar{\rho }$ as $\lambda_{c}=\frac{2\pi }{\kappa_{c}}$, which diverges at $\beta \bar{\rho }\to {\left(\beta \bar{\rho }\right)}_{c}\text{so}\lambda_{c}\to \infty$ and approaches λ_*c*_ → 4 for $\beta \bar{\rho }\to \infty$. In conclusion, our mean-field analysis allows us to predict the onset of the instability of the homogeneous steady state in dependence on density *ρ* and sensitivity *β* as well as the wave length of the observed patterns without the need for computer simulations, as seen in Fig 6.

### Explaining invasion plasticity of breast cancer

Progression of cancer depends on changes at the individual cell level and interactions of healthy and malignant cells. Various CA models have been suggested for selected aspects of cancer progression (see e.g. [27, 28]). Here, we shed light on the invasion plasticity of growing tumours. Solid tumours have been observed to switch between jammed (immobile, glass-like), highly correlated collective movement (active nematic phase) and single-cell-disseminating, uncorrelated (gas-like) states, which is often referred to as invasion plasticity [29–31]. These behaviours result from an interplay between cell-cell and cell-ECM interactions, which have been recently studied using a BIO-LGCA model [16]. Computational simulations showed remarkable similarity to the experimental mammary gland carcinomas. Here, we generalise the computational results in [16] by deriving an analytical theory for the observed phase transitions.

We will consider a 1D system (akin to cells moving inside an ECM duct). In this case, cells can only move in the positive or negative direction, thus the velocity channels are given by *c*_1_ = 1 and *c*_2_ = −1. As in the original model, we incorporate the effect of cell-cell adhesion as attractive and velocity alignment interactions. Additionally, we mimic steric interactions between cells by an increased tendency of cells to remain in rest channels if their neighbours occupy rest channels as well. However, while in the aforementioned elementary models the relative strengths of these interactions were assumed to be fixed, and their absolute strengths were varied by a single parameter, we here treat these mechanisms as independent, to obtain the full spectrum of possible migration modes. Our model considers *b* = 2 velocity channels and *a* rest channels, for a total of *K* = 2 + *a* channels. Under these assumptions, the BIO-LGCA transition probabilities are given by

$$\begin{array}{c} P(s\to s^{O}|s_{N})=\frac{\delta_{n(s),n(s^{\prime })}}{Z}\text{exp}\;[E(s,s^{\prime },s_{N})]. \end{array}$$ (35)

The energy function is divided into three contributions

$$\begin{array}{c} E=E_{\text{agg}}+E_{\text{align}}+E_{\text{rest}}. \end{array}$$

The aggregation energy is given by the logistic density gradient, $E_{\text{agg}}=\beta_{\text{agg}}j(s^{O})g_{\text{agg}}(s_{N})$, where $j(s^{O})=\sum_{j=1}^{b}s_{j}^{O}c_{j}$ is the post-interaction flux, and the logistic density gradient

$$\begin{array}{c} g_{\text{agg}}(s_{N})=\sum_{j=1}^{b}n(s(r+c_{j}))[1-\frac{n(s(r+c_{j}))}{n_{\text{crit}}}]c_{j}, \end{array}$$ (36)

where *n*_crit_ is a parameter controlling the maximum density of cell aggregates, thus modelling the homeostatic cell density.

The alignment energy corresponds to the collective cell migration interaction (Eq 18), here given by $E_{\text{align}}=\beta_{\text{align}}j(s^{O})g_{\text{align}}(s_{N})$, where the post-interaction flux is as before and the neighbourhood flux

$$\begin{array}{c} g_{\text{align}}(s_{N})=\sum_{j=1}^{b}j(s(r+c_{j})). \end{array}$$ (37)

Finally, the resting energy is given by $E_{\text{rest}}=\beta_{\text{rest}}n_{\text{rest}}(s^{O})n_{\text{rest}}(s_{N})$, where the local resting cell density is $n_{\text{rest}}(s^{O})=\sum_{j=b+1}^{K}s_{j}^{O}$ and the neighbourhood resting cell density is $n_{\text{rest}}(s_{N})=\sum_{j=1}^{b}n_{\text{rest}}(s(r+c_{j}))$.

To facilitate further analysis, we assume that the density is low, and that there is at most one cell at each node. Then, under the mean-field approximation the dynamics of the BIO-LGCA model is given by the lattice-Boltzmann (LBE) equation

$$\begin{array}{c} f_{j}(r+c_{j},k+1)=\rho (r,k)T_{j}, \end{array}$$ (38)

where *f*_*j*_(*r*, *k*) ≔ 〈*s*_*j*_(*r*, *k*)〉, $\rho \left(r,k\right)=\sum_{j=1}^{K}f_{j}\left(r,k\right)$, and $T_{j}≔P(s_{j}^{O}=1|s_{N})$ is the single particle probability given by

$$\begin{array}{c} T_{j}=\{\begin{array}{cc} \frac{1}{Z}\;\text{exp}\;[\beta_{\text{agg}}c_{j}g_{\text{agg}}+\beta_{\text{align}}c_{j}g_{\text{align}}] & j\leq 2\\ \frac{1}{Z}\;\text{exp}\;[\beta_{\text{rest}}n_{\text{rest}}] & j>2. \end{array} \end{array}$$ (39)

The homogeneous steady state of the LBE, where the density, $\bar{\rho }$, occupation of velocity channels, *f*_*v*_, and rest channels, *f*_*r*_, is constant and identical in every lattice node is given by

$$\begin{array}{cc} \bar{\rho } & =2f_{v}+af_{r}, \end{array}$$ (40)

$$\begin{array}{cc} f_{v} & =\frac{\bar{\rho }}{2+a\Pi_{r}}, \end{array}$$ (41)

where

$$\begin{array}{c} \Pi_{r}=\text{exp}(2\beta_{\text{rest}}af_{r}). \end{array}$$ (42)

The steady states can now be calculated numerically. Subsequently, We linearise the LBE around the numerically obtained steady states, and apply a discrete Fourier transform with respect to the spatial coordinates to eliminate dependencies on spatial increments. Then, the stability of the steady states is given by the eigenvalues, Λ, of the Boltzmann propagator matrix

$$\begin{array}{c} \Gamma_{m,n}\left(q\right)=e^{-ic_{m}q}[T_{m}+\sum_{\ell =1}^{2}e^{ic_{\ell }q}\frac{\partial }{\partial f_{n}(r+c_{\ell },k)}f_{m}(r+c_{m},k+1)], \end{array}$$ (43)

where *q* ∈ [0, 2*π*) is the wavevector, and *f*_*m*_(*r* + *c*_*m*_, *k* + 1) is given by the LBE.

**Fig 7:**
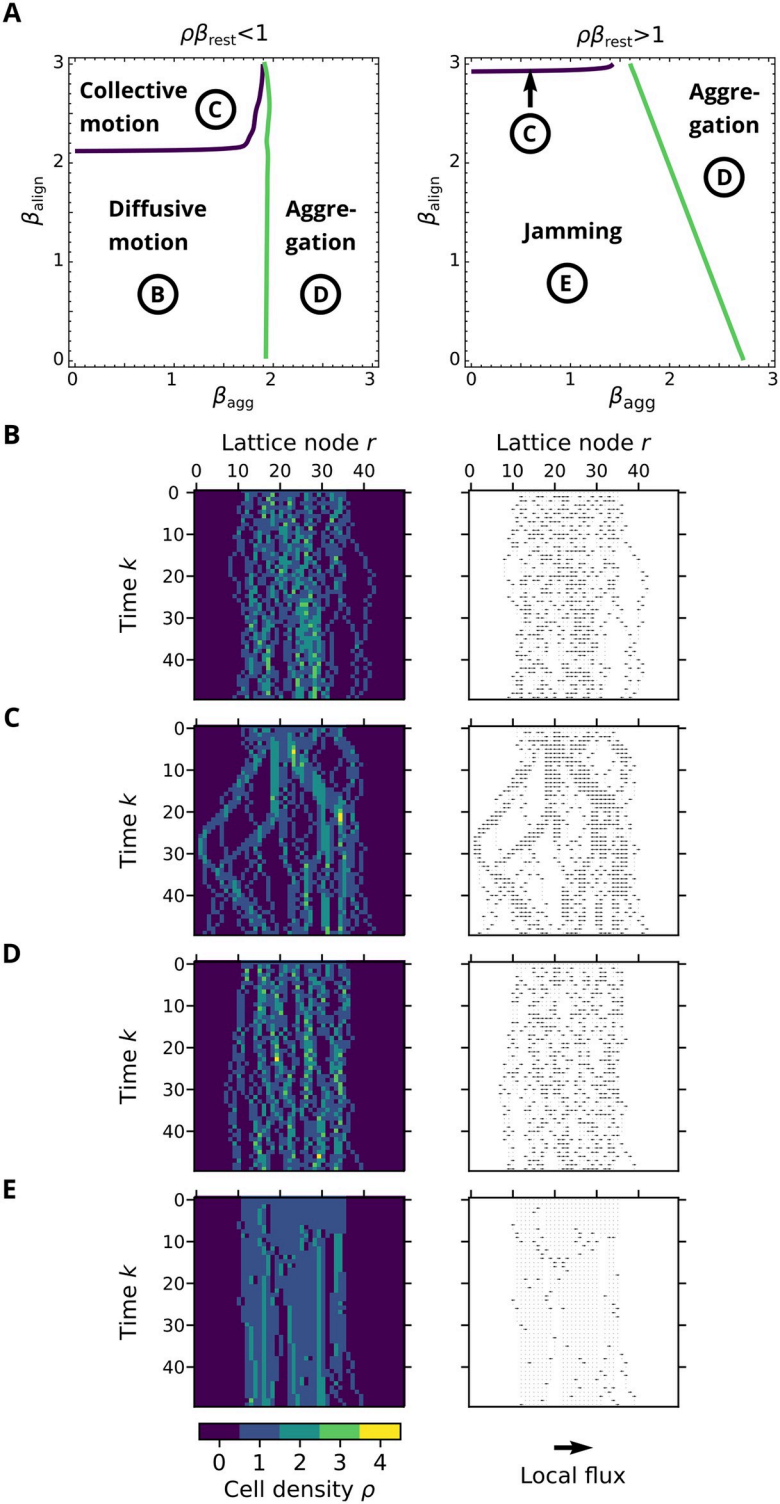
Migration modes in the BIO-LGCA model. A: Phase diagram for low (left) and high (right) values of *β*_rest_. B-E: Snapshots of the different phases of cell density (left) and local flux (right): B: diffusive motion, if all sensitivities *β* are low: cells interact weakly with one another and with the ECM; C: collective motion for high *β*_align_; D: pattern formation for high *β*_agg_; E: jammed state for high *β*_rest_.

The maximum of the modulus of the Boltzmann propagator eigenvalues, |Λ| gives information on the wavelength $\lambda =\frac{2\pi }{q}$ of spatial patterns observed in the model, while the argument of the eigenvalues, arg(Λ), defines the propagation velocity of these patterns. Calculating these numerically, we find the phase space shown in Fig 7.

We can identify four distinct regions in the parameter space.

1. Diffusive (gas-like) phase. In this region, cells move freely and diffuse within the ECM duct. In the phase space, this corresponds to the region of the parameter space with low *β*_align_, *β*_agg_, and *β*_rest_ values, see Fig 7B.
2. Collective motion (active nematic) phase. This is the region with high *β*_align_. In this region, cells move collectively into the same direction, see Fig 7C.
3. Aggregation phase. In this region, cells arrange themselves in static clusters and form cellular patterns within the duct. This corresponds to the region with high *β*_agg_, see Fig 7D.
4. Jammed (glass-like) phase. This corresponds to the regime with high *β*_rest_. In this region, cells neither form patterns nor do they move collectively. However, almost all cells are in a resting channel and the dynamics is frozen, similar to a crystalline solid, see Fig 7E.

In conclusion, we can reproduce the prime modes of collective migration using three mechanisms: alignment of velocities, aggregation and an inhibition of motion by non-migratory cells. We here treated these mechanisms as independent, however, in reality, they are the result of a complex interplay of cell-cell adhesion, cellular contractility, and intercellular signaling. Identifying the regulation of these cellular properties is a topic of ongoing research.

## Discussion

In contrast to “continuum systems” and their canonical description with partial differential equations, there is no standard model for describing interactions of discrete objects, particularly interacting and migrating discrete biological cells. In this paper, the BIO-LGCA is proposed as a lattice-based model class for a spatially extended system of interacting cells. We provided various examples of BIO-LGCA models for homogeneous cell populations, i.e. cells are assumed to be of the same phenotype and not to change their behaviour. Examples include haptotaxis, chemotaxis, contact guidance and collective migration. However, the BIO-LGCA idea can be expanded to heterogeneous populations and environments, e.g. cells may dynamically regulate their adhesivities and/or may interact with a heterogeneous non-cellular environment [32]. BIO-LGCA models have already been used in the study of several biological processes including angiogenesis [33], bacterial rippling [34], active media [35], epidemiology [36] and various aspects of tumour dynamics [16, 28, 32, 37–39].

BIO-LGCA models are appropriate for low and moderate cell densities. For higher densities, e.g. in epithelial tissues, cell shape may matter and other models, such as the Cellular Potts model, might be better choices (see [2, 40] for reviews of on- and off-lattice models). It is also important to be aware of lattice artefacts due to the spatial discretisation inherent in every cellular automaton model, e.g. the checkerboard artefact (cp. [41]). In two spatial dimensions, the hexagonal lattice possesses less artefacts than the square lattice. A major advantage of BIO-LGCA models compared to other on- and off-lattice cell-based models for interacting cell systems, such as interacting particle systems, e.g. [42], asynchronous cellular automata, e.g. [43–45], further cell-based models [46] or systems of stochastic differential equations [47], is their computational efficiency, and their synchronicity and explicit velocity consideration, which enables the modelling of moderately packed cell collectives while minimising model artefacts.

The BIO-LGCA framework facilitates theoretical analysis of collective behaviour [41]. In many cases, the collective behaviour of the BIO-LGCA can be analysed using reasonable approximations, such as a spatial mean-field description resulting on a partial differential equation [41, 48–50]. Collective behaviour at the tissue scale includes cell density patterns and the dynamics of moving cell fronts [51, 52]. Cell density patterns can often be assessed experimentally and provide, therefore, a means to relate BIO-LGCA model predictions to experimental observations.

Meanwhile, off-lattice models formulated as stochastic differential equations for moving and interacting cells have been derived from individual-based BIO-LGCA models [53]. Individual-based BIO-LGCA are lattice-based models that allow to distinguish and track individual cells which is not possible in classical LGCA models (e.g. [48]). We have also defined a “boson-type” LGCA which contrary to the BIO-LGCA does not possess an exclusion principle with respect to the velocity channels and facilitates mathematical analysis [54]. To model cell migration involving steric effects, the model has to be extended to discourage individuals moving into the direction of steeply increasing cell density gradients.

The BIO-LGCA modelling strategy is “modular” starting from “basic model moduli”, which include those explored in this paper such as alignment, attraction, contact guidance, hapto- and chemotaxis. Coupling the moduli is required to design models for complex biological problems. The focus of future activities is the analysis of further model combinations for selected biological problems, which are not necessarily restricted to cells but could also comprise interactions at subcellular and tissue scales. The resulting multi-scale models will contain a multitude of coupled spatial and temporal scales and will impose significant challenges for their analytic treatment.
